# Behavioral Predictors of Intentional and Unintentional Nonadherence to Antiretroviral Therapy and Their Implications for Virological Failure Among People with HIV in Taiwan

**DOI:** 10.3390/v17101375

**Published:** 2025-10-14

**Authors:** Su-Han Hsu, Chien-Chun Wang, Yung-Feng Yen, Tsen-Fang Yen, Po-Tsen Yeh, Hsin-Hao Lai

**Affiliations:** 1 Department of Pharmacy, Taipei City Hospital Yangming Branch, Taipei 111, Taiwan; a3772@tpech.edu.tw; 2 School of Pharmacy, College of Pharmacy, Taipei Medical University, Taipei 110, Taiwan; 3 Department of Exercise and Health Sciences, University of Taipei, Taipei 111, Taiwan; 4 Division of Infectious Diseases, Department of Internal Medicine, Taipei City Hospital, Linsen, Chinese Medicine, and Kunming Branch, Taipei 108, Taiwan; das32@tpech.gov.tw (C.-C.W.); t0387@tpech.gov.tw (P.-T.Y.); 5 Division of Infectious Disease, Department of Internal Medicine, Taipei City Hospital, Heping Branch, Taipei 100, Taiwan; yfyen1@gmail.com; 6 Department of Nursing, Taipei City Hospital, Linsen, Chinese Medicine, and Kunming Branch, Taipei 108, Taiwan; b2348@tpech.gov.tw; 7 Division of Infectious Disease, Department of Internal Medicine, Taipei City Hospital, Yangming Branch, Taipei 111, Taiwan; 8 School of Medicine, College of Medicine, National Yang Ming Chiao Tung University, Taipei 112, Taiwan

**Keywords:** medication adherence, psychosocial factors, recreational drug use, single-tablet regimen, viral suppression, socioeconomic status, HIV disclosure, people with HIV (PWH)

## Abstract

Adherence to antiretroviral therapy (ART) is critical for HIV management and sustained virological suppression. Differentiating intentional from unintentional nonadherence is essential for developing tailored interventions, yet evidence from Asian populations remains limited. A cross-sectional study of 846 people with HIV (PWH) in northern Taiwan assessed ART adherence using the MARS-5 scale. Participants were categorized into good, unintentional, or intentional non-adherence groups. Logistic regression identified associated behavioral and psychosocial factors. Recreational drug use and younger age were independently linked to both unintentional and intentional poor adherence. Higher income and the use of single-tablet regimens were protective against intentional nonadherence, whereas disclosure of HIV status to a partner and an unsuppressed viral load were significantly associated with intentional nonadherence. Reported reasons included being too busy, emotional distress, and running out of medication. These findings suggest that intentional and unintentional nonadherence represent distinct behavioral patterns, with intentional lapses more strongly linked to virological failure. Addressing substance use, simplifying regimens, and providing psychosocial support after disclosure are essential to optimize adherence and achieve UNAIDS 2030 targets.

## 1. Introduction

Medication adherence—defined as the extent to which patients take medications as prescribed—is a critical determinant of clinical outcomes, particularly in chronic disease management [1]. Poor adherence contributes to an estimated 125,000 deaths annually in the United States and nearly USD 300 billion in avoidable healthcare costs [2]. Notably, improving adherence may yield greater health benefits than advances in pharmacotherapy [3]. In the context of HIV, the advent of antiretroviral therapy (ART) has transformed the disease into a manageable chronic condition. However, ART adherence remains a global challenge. A meta-analysis reported adherence rates ranging from 53% to 84% [4]. In Taiwan, the National Health Insurance (NHI) program fully subsidizes the cost of antiretroviral therapy (ART), ensuring that all people living with HIV can access treatment without financial burden. Despite comprehensive interventions over the past two decades that enabled the country to achieve the UNAIDS 90-90-90 targets in 2021, poor ART adherence remains a persistent issue. As of August 2025, 36,363 people were living with HIV (PWH) [5] and a 2024 survey conducted in northern Taiwan revealed that approximately 11% of PWH exhibited poor adherence to ART [6], underscoring persistent gaps in adherence behavior.

ART adherence is pivotal to clinical success in PWH. Suboptimal ART adherence leads to virologic failure, CD4 cell decline, and progression to AIDS, thereby increasing the risk of AIDS-defining illnesses (ADIs), mortality, and healthcare costs [7,8,9]. Specifically, when CD4 counts drop from 500 to 50 cells/μL, the incidence of ADIs increases approximately 25-fold [7], and the ADI-related mortality rate reaches up to 16.9% in Taiwan [8]. Moreover, the presence of ADIs nearly doubles annual healthcare expenditures—a 99% increase—compared to PWH without ADIs, posing additional clinical and economic challenges [9]. Conversely, regular ART use reduces morbidity, mortality, HIV transmission, and treatment resistance [10]. Therefore, achieving the UNAIDS 2030 target of ending the ADIS epidemic requires sustained efforts to improve ART adherence.

Poor medication-taking behavior can be categorized as either intentional or unintentional [11]. Intentional non-adherence involves a conscious decision to deviate from prescribed regimens, often influenced by beliefs, concerns, or stigma [12,13]. Unintentional non-adherence, by contrast, stems from forgetfulness, misunderstanding, or logistical barriers [14,15]. Differentiating these types is essential for designing effective, tailored interventions. Prior studies have explored these distinctions among PWH. Intentional non-adherence has been linked to concerns about side effects, negative beliefs about medications, and substance use—particularly in the context of chemsex and interactive toxicity beliefs [12,13,16,17,18]. Unintentional non-adherence is more often associated with younger age, depression, and practical challenges [12,13]. HIV-related stigma further compounds these issues, contributing to psychological distress and reduced adherence [19,20,21].

Despite growing evidence, the sociocultural determinants of intentional and unintentional non-adherence remain underexplored in Asian populations. This study aims to assess the associations of sociodemographic characteristics, recreational drug use, and depressive symptoms with both types of poor adherence among Taiwanese PWH. Beyond adherence behavior per se, distinguishing intentional from unintentional nonadherence is clinically important, as only sustained and deliberate lapses are likely to drive virological failure and resistance. Identifying these behavioral predictors provides critical insights for optimizing personalized ART strategies and contributes to the broader goal of patient-centered HIV care.

## 2. Materials and Methods

### 2.1. Study Population and Procedures

This cross-sectional study was conducted at a hospital in Taipei, Taiwan, between December 2018 and November 2020. Individuals aged 18 years or older who had been diagnosed with HIV and were receiving ART were invited to participate during their routine clinic visits. After providing written informed consent, participants completed a well-structured questionnaire and interviewed if needed, which required approximately 15 min to complete. As compensation for their time, each participant received a US$3 coupon. The study was approved by the Institutional Review Board of Taipei City Hospital (approval number: TCHIRB-10612120).

### 2.2. Study Instruments

Participants completed a questionnaire that collected information on demographic characteristics (including age, sex, sexual orientation, body mass index, income, education level, and living status), HIV-related clinical status (years of HIV diagnosis, duration of antiretroviral therapy, viral load, CD4 count, ART regimen classification, and disclosure of HIV status), health behaviors (such as smoking, alcohol consumption, and recreational drug use within the past three months. Recreational drug use included substances commonly reported among Taiwanese PWH, such as ketamine, methamphetamine, and others. individuals reporting such use were defined as active drug users), comorbidities (including history of sexually transmitted disease, hepatitis B virus [HBV], and hepatitis C virus [HCV]) acquired, and depressive symptoms. Depressive symptoms were assessed using the Center for Epidemiologic Studies Depression Scale (CES-D), which has been validated in Taiwan [22]. A cut-off score of 16 or higher indicated the presence of depressive symptoms. In this study, the internal consistency was good (Cronbach’s α = 0.836).

The Medication Adherence Report Scale (MARS-5), a self-reported instrument for assessing medication adherence, has demonstrated strong reliability and validity in previous studies [13,23]. Its use among Taiwanese people with HIV has also been documented [24]. In this study, the Chinese version of the MARS-5 was applied, consisting of one item assessing unintentional non-adherence and four items assessing intentional non-adherence [25]. The internal consistency of the scale in our sample was acceptable (Cronbach’s α = 0.799). The item representing unintentional non-adherence was: (i) “I forget to take my medications.” The items reflecting intentional non-adherence were: (ii) “I change the dose of my medications,” (iii) “I temporarily stop taking my medications,” (iv) “I decide to miss a dose,” and (v) “I take less than prescribed.” Responses were measured on a 5-point Likert scale, with scores ranging from 1 (“always”) to 5 (“never”). Total scores ranged from 5 to 25, with higher scores indicating better adherence. Participants with a total score of 25 were categorized as having good adherence. Any item scored below 5 was considered indicative of non-adherence. Specifically, a score below 5 on item (i) was classified as unintentional non-adherence, while scores below 5 on items (ii) to (v) were considered intentional non-adherence. In addition to the validated MARS-5 instrument, participants reporting non-adherence were asked to provide reasons in an open-ended format. If participants responded with a score below 5 on any MARS item, the researchers conducted follow-up inquiries to collect additional information regarding the reasons for the reported non-adherence.

### 2.3. Statistical Analysis

Based on their responses to the MARS items, participants were categorized into three groups: good adherence (Group 1), defined as a total MARS score of 25; unintentional poor adherence (Group 2), defined as a score below 5 on item (i) only, with scores of 5 on items (ii) through (v); and intentional poor adherence (Group 3), defined as scores below 5 on any of items (ii) through (v), with or without unintentional poor adherence.

Demographic characteristics were compared across the three adherence groups. Categorical variables are presented as frequencies and percentages, while continuous variables are expressed as means with standard deviations (SD). Differences between groups were analyzed using analysis of variance (ANOVA) for continuous variables and the chi-square test for categorical variables. Post hoc comparisons following ANOVA were conducted using the Bonferroni correction to identify specific group differences. A *p*-value of < 0.05 was considered statistically significant.

Logistic regression was used to analyze the odds ratios for unintentional and intentional poor adherence compared to good adherence. Variables with a *p*-value ≤ 0.10 in univariate analyses were included in the multivariate logistic regression model using a backward elimination approach. As each non-adherence item may have multiple reasons, the frequencies of reasons for unintentional and intentional non-adherence were reported as counts. All statistical analyses were carried out using SPSS software, version 22.0 (SPSS Inc., Chicago, IL, USA).

## 3. Results

### 3.1. Characteristics of PWH by 3 Different Adherence Groups

A total of 846 people living with HIV (PWH) were included in the analysis. The majority were male (98.90%), with a mean age of 37.46 years with standard deviation (SD) of 8.89. On average, participants had been diagnosed with HIV for 9.05 years (SD = 5.54) and had been receiving ART for 7.73 years (SD = 5.57). Most participants identified as homosexual (92.9%), and 76% had attained a university degree or higher. 64.9% reported a history of sexually transmitted diseases. Additionally, 14.4% had used recreational drugs in the past three months. Most participants (90.7%) achieved viral suppression, and 39.6% exhibited depressive symptoms, defined as a CES-D score ≥16.

Among the participants, 521 exhibited good adherence (Group 1), 190 had unintentional non-adherence (Group 2), and 135 had intentional non-adherence with or without unintentional non-adherence (Group 3). Within Group 3, 110 participants exhibited both intentional and unintentional non-adherence, while the remaining 25 reported only intentional non-adherence. Participants in Group 1 were older (mean age 38.45 vs. 34.67 years) and had a longer duration since HIV diagnosis (9.55 vs. 7.83 years) compared to those in Group 3. A higher proportion of participants in Group 1 had a university degree or above compared to Group 3 (79.1% vs. 68.9%). Additionally, both Group 1 and Group 2 had a greater proportion of participants with high income levels compared to Group 3 (51.8% vs. 34.1%; 50% vs. 34.1%, respectively).

Conversely, the prevalence of recreational drug use was lower in Group 1 compared to Group 2 and Group 3 (10.0% vs. 18.9% and 25.2%, respectively). Sharing HIV status with partner was less common in Group 1 than in Group 3 (63.0% vs. 74.8%). Group 1 had a higher proportion of participants with CD4 counts ≥200 cells/μL than Group 3 (99% vs. 95.6%). Furthermore, a greater proportion of participants in Group 1 and Group 2 achieved viral suppression compared to Group 3 (95.0% vs. 74.1% and 90.5% vs. 74.1%, respectively). Additional details on the characteristics of PWH across the three adherence groups are presented in Table 1.

### 3.2. Factors Associated with Adherence

Logistic regression analysis was arranged to determine the factors associated with adherence which was showed in Table 2. Univariate analysis identified several factors associated with unintentional non-adherence. Older age (Odds ratio [OR] = 0.978, *p* = 0.024) and longer duration since HIV diagnosis (OR = 0.967, *p* = 0.031) appeared to be protective, whereas recent recreational drug use within the past three months (OR = 2.108, *p* = 0.002), and having a detectable viral load (OR = 1.992, *p* = 0.031) were associated with a higher likelihood of unintentional non-adherence. Multivariate analysis confirmed that age remained a protective factor (aOR = 0.981, *p* = 0.048), while recent recreational drug use (aOR = 1.961, *p* = 0.005) were significantly associated with increased odds of unintentional non-adherence.

Several factors were associated with intentional non-adherence in the univariate analysis. Protective factors included older age (OR = 0.950, *p* < 0.001), longer duration since HIV diagnosis (OR = 0.943, *p* = 0.001), higher educational attainment (OR = 0.586, *p* = 0.007), higher income level (OR = 0.480, *p* < 0.001), being in a romantic relationship (OR = 0.646, *p* = 0.032), absence of depressive symptoms (CES-D <16) (OR = 0.649, *p* = 0.027), CD4 count ≥200 cells/μL (OR = 0.208, *p* = 0.011), and the use of single-tablet regimen (STR) (OR = 0.603, *p* = 0.030). In contrast, recent recreational drug use within the past three months (OR = 3.036, *p* < 0.001), disclosure of HIV status to a romantic partner (OR = 1.748, *p* = 0.010), and an unsuppressed viral load (OR = 6.663, *p* < 0.001) were associated with increased odds of intentional non-adherence. After adjustment in multivariate analysis, older age (aOR = 0.969, *p* = 0.048), higher income level (aOR = 0.583, *p* = 0.014) and the use of STR (aOR = 0.452, *p* = 0.003) remained significant protective factors. Conversely, recent recreational drug use (aOR = 2.920, *p* < 0.001), disclosure of HIV status to a romantic partner (aOR = 1.639, *p* = 0.043), and having an unsuppressed viral load (aOR = 5.696, *p* < 0.001) were independently associated with higher odds of intentional non-adherence.

Logistic regression analysis indicated that poor adherence among PWH was associated with recreational drug use in the past three months, whereas older age appeared to be protective in both groups. A higher income level and the use of a single-tablet regimen were protective against intentional but not unintentional poor adherence. Furthermore, disclosure of HIV status to a romantic partner and lack of viral suppression were associated with intentional poor adherence. Importantly, participants with intentional nonadherence were significantly more likely to exhibit an unsuppressed viral load, underscoring the clinical consequences of deliberate treatment lapses.

### 3.3. Reasons for Medication Non-Adherence

Among the 190 participants in Group 2, the most frequently reported reason for unintentional non-adherence was being “Being busy with work/life” (53.68%) (Table 3). Other common reasons included “forgot to bring medication” (15.79%), “missed dose due to sleep” (14.21%), and “forgot whether the medication had been taken” (7.37%). In Group 3, 43 participants did not provide a response or did not report a specific reason for intentional non-adherence. Among those who did, the most cited reason was “ran out of medication” (25.00%), followed by “experienced side effects” (14.81%), “in a poor emotional state” (14.81%), and “did not want to take medication” (12.59%).

## 4. Discussion

This study investigated factors associated with suboptimal ART adherence among PWH in Taiwan, distinguishing between unintentional and intentional non-adherence. The results identified recent recreational drug use and younger age as significant risk factors for both types of non-adherence, while higher income and STRs were protective against intentional non-adherence. In contrast, disclosure of HIV status to a partner and having a detectable viral load were identified as risk factors for intentional non-adherence.

The prevalence of recent recreational drug use among participants (14.4%) was substantially higher than the general population in Taiwan which around 1.46% lifetime use [26], suggesting a concentrated burden among PWH. This finding aligns with international studies in the United States and South Africa showing that single or combined substance use is strongly associated with ART non-adherence [27,28]. Intentional non-adherence may be driven by “interactive toxicity beliefs”—the perception that combining ART with recreational drugs is harmful—leading individuals to skip doses. Unintentional non-adherence, meanwhile, may result from cognitive impairment or forgetfulness induced by substance use [29]. Table 3 supports this interpretation: among those reporting unintentional nonadherence, common reasons included “missed dose due to sleep” (14.21%) and “forgot to take medication due to recreational drug use” (4.74%), reflecting the disorganizing effects of drug use. Conversely, reports of “intentionally skipped due to recreational drug use” provide direct evidence for interactive toxicity beliefs as a driver of intentional nonadherence.

Younger age was another significant predictor of non-adherence. A meta-analysis of adherence among older HIV-infected individuals found that older adults had significantly lower odds of nonadherence, with approximately a 27% reduction in risk compared to younger counterparts [30]. Similarly, a systematic review of socio-demographic determinants of ART adherence indicated that although results across studies were mixed, several reported a positive association between increasing age and improved adherence [31]. Several explanations may account for this relationship. Older individuals are more likely to achieve psychological acceptance of their HIV diagnosis [32] and the chronic nature of the disease [30], and stigma tends to decline with age [33]. They also often demonstrate greater maturity, more stable lifestyles, and consistent daily routines, which reduces the likelihood of forgetfulness or missed doses. In addition, accumulated experience with healthcare may enhance their understanding of ART benefits, and many older patients have transitioned to regimen with lower pill burden and fewer adverse effects [34]. In contrast, younger PWH often face irregular schedules, social pressures, and higher engagement in risk behaviors such as substance use [35,36]. These lifestyle factors may contribute to both intentional and unintentional non-adherence. Table 3 supports this interpretation, showing that reasons such as being “being busy with work/life” (53.68%), “forgot whether medicine was taken” (7.37%), “daily routine did not match schedule,” “Fatigue from work/life” (3.68%), and “ran out of medication” (18.52%) were common. These findings underscore the need for age-tailored interventions that address both behavioral and psychosocial barriers to adherence. These findings imply that adherence support may need to be age-specific, with younger PWH requiring more behavioral interventions to prevent virological failure.

Higher income was associated with reduced intentional non-adherence. A systematic literature review conducted in high-income countries reported that lower socioeconomic status was linked to poorer virological and immunological responses, as well as a higher risk of nonadherence to ART, with no evidence suggesting a protective effect of socioeconomic disadvantage [37]. Patients with lower income may face financial barriers to consistent healthcare access, competing life priorities, and unstable living conditions, all of which undermine adherence. Furthermore, STRs were linked to improved adherence, likely due to simplified dosing schedules and reduced pill burden. These results are consistent with prior studies showing that STRs enhance treatment satisfaction and viral suppression [38,39,40]. Table 3 further supports this point: reasons such as “did not want to take medication” (12.59%) and “too many medications” (1.48%) suggest that regimen fatigue may contribute to nonadherence, which STRs could help mitigate.

Disclosure of HIV status to a partner and having a detectable viral load were unexpectedly associated with intentional nonadherence. The majority of participants in our study were men who have sex with men (MSM), which closely reflects the national epidemiological trend in Taiwan, where MSM account for approximately 80% of newly reported HIV infections in recent years (Taiwan Centers for Disease Control) [5]. Thus, the characteristics of our sample are consistent with the broader population of people living with HIV in Taiwan. Although disclosure is often considered a pathway to social support, its impact largely depends on the partner’s reaction. According to the Disclosure Process Model, negative responses can exacerbate stigma and emotional distress, thereby reducing adherence [41,42]. Table 3 provides further insight: 14.81% of intentional nonadherence cases were attributed to being “in a poor emotional state,” and 2.96% to being “afraid that taking medication would reveal HIV status.” These reasons may reflect internalized stigma or psychological burden following disclosure, particularly when it is met with rejection or judgment.

An unsuppressed viral load was independently associated with intentional—but not unintentional—nonadherence. Intentional nonadherence behavior such influence by toxicity behavior that mentioned above were strongly linked to virologic failure [27,28]. In contrast, unintentional lapses such as forgetting a dose have a weaker and less consistent impact, as occasional missed doses may still meet the WHO-recommended 90–95% adherence threshold [43,44]. Therefore, occasional unintentional missed doses may still fall within this adherence threshold. Moreover, the pharmacokinetic “forgiveness” of modern antiretroviral regimens can buffer such sporadic lapses and sustain viral suppression despite imperfect adherence [45]. These findings indicate that intentional nonadherence is a stronger predictor of persistent viremia and resistance, highlighting the need for interventions targeting its underlying behavioral drivers.

Depression is a prevalent comorbidity among people living with HIV, as highlighted by a recent systematic review and meta-analysis reporting a high prevalence of depressive symptoms in this population [46]. Although the study did not directly examine the effect of depression on ART adherence, previous research has consistently suggested that depressive symptoms can negatively influence medication-taking behaviors [47], In our multivariate analysis, however, this association was not confirmed. One possible explanation is that the CES-D cutoff of 16 may capture transient mood disturbances rather than clinically diagnosed depression, thereby reducing specificity. In addition, our study population—characterized by younger age, high viral suppression rates, and universal ART coverage in Taiwan—may have mitigated the impact of depression on adherence. Furthermore, after adjusting for variables such as recreational drug use, income, and viral load, the independent effect of depressive symptoms may have been attenuated due to confounding or mediation. Despite this, psychological support remains important in HIV care, particularly for subgroups at higher risk, such as those with substance use or socioeconomic disadvantage.

Finally, the self-reported reasons summarized in Table 3 integrate and reinforce our main findings. Unintentional nonadherence was largely driven by lifestyle demands (“being busy with work/life,” 53.68%), forgetfulness, or disruptions from recreational drug use, reflecting the vulnerability of younger PWH with irregular routines and substance involvement. In contrast, intentional nonadherence often reflected psychosocial and structural barriers, such as “ran out of medication” (18.52%), “experienced side effects” (14.81%), “poor emotional state” (14.81%), or fear of HIV disclosure (2.96%). These patterns align with our quantitative analyses showing that recreational drug use, lower income, detectable viral load, and disclosure were risk factors, whereas age, STR, and higher income were protective. Together, these findings highlight that adherence is shaped by both cognitive lapses and deliberate decisions influenced by social and emotional contexts. Interventions must therefore address practical barriers through integration with harm reduction programs, regimen simplification and reminder systems, while simultaneously targeting psychosocial determinants such as stigma, mental health, and partner dynamics to optimize ART outcomes.

Several limitations should be acknowledged. First, the cross-sectional design precludes causal inference; associations observed between behavioral factors and adherence cannot confirm temporal directionality. Second, self-reported data on adherence and recreational drug use may be subject to recall bias or social desirability bias, potentially underestimating non-adherence or drug use prevalence. Third, the study did not quantify alcohol consumption, limiting the ability to assess its dose-dependent impact on adherence [48]. Fourth, the sample was drawn from a single hospital in northern Taiwan, which may limit generalizability to other regions or healthcare settings. In addition, the data were collected between 2018 and 2020. Although there was a delay in publication, the identified factors such as recreational drug use and depression remain clinically relevant. Lastly, while the study included multiple psychosocial variables, unmeasured factors such as stigma, trauma history, or executive functioning may also influence adherence behaviors and warrant further investigation.

Nevertheless, the study’s strengths help mitigate these limitations. The large sample size (*n* = 846) enhances statistical power and internal validity. The use of validated instruments (CES-D and MARS-5) ensures measurement reliability. Importantly, the distinction between intentional and unintentional non-adherence offers a novel behavioral framework rarely examined in Asian contexts. Our findings also reinforce that both intentional and unintentional non-adherence may be influenced by multiple factors. In clinical practice, paying close attention to patient-reported outcomes is crucial for identifying ART adherence problems and tailoring interventions accordingly. Additionally, follow-up inquiries added qualitative depth, and the focus on Taiwanese PWH contributes valuable cultural insights to the global HIV literature.

## 5. Conclusions

This study identified distinct determinants of ART nonadherence among PWH in Taiwan. Recreational drug use and younger age increased the risk of both unintentional and intentional nonadherence, while higher income and single-tablet regimens protected against intentional nonadherence. Disclosure of HIV status and detectable viral load were uniquely associated with intentional nonadherence, highlighting the psychosocial burden that may follow disclosure. Importantly, only intentional nonadherence was strongly linked to viral non-suppression, underscoring its critical role as a behavioral driver of virological failure. These findings suggest that interventions should move beyond general adherence promotion to address recreational drug use, regimen simplification, and post-disclosure support. By differentiating between intentional and unintentional lapses, our results provide behavioral predictors that can inform personalized ART strategies and contribute to sustaining viral suppression in pursuit of the UNAIDS 2030 goals.

## Figures and Tables

**Table 1:** Characteristics of the PWH, by adherence.

Characteristics		No. (%) of Subjects			
	Total *n* = 846	Full Adherence, *n* = 521 (group 1)	Unintentional Non-Adherence, *n* = 190 (Group 2)	Intentional Non-Adherence, *n* = 135 (Group 3)	*p* Value
**Socio-demographics**					
Age, yr					
Mean (SD)	37.460 (8.892)	38.45 (9.258)	36.72 (8.235)	34.67 (7.611)	<0.001
Diagnosed, yr					
Mean (SD)	9.047 (5.544)	9.553 (5.664)	8.527 (5.408)	7.828 (5.022)	0.002
Treatment duration, yr					
Mean (SD)	7.733 (5.577)	8.062 (5.735)	7.345 (5.293)	7.009 (5.373)	0.081
Sex					0.87
Female	9 (1.1)	5 (1.0)	2 (1.1)	2 (1.5)	
Male	837 (98.9)	516 (99.0)	188 (98.9)	133 (98.5)	
Sex orientation					0.846
Non-Homosexual	60 (7.1)	39 (7.5)	12 (6.3)	9 (6.7)	
Homosexual	786 (92.9)	482 (92.5)	178 (93.7)	126 (93.3)	
Education level completed					0.022
≤High school	203 (24.0)	109 (20.9)	52 (27.4)	42 (31.1)	
University or above	643 (76.0)	412 (79.1)	138 (72.6)	93 (68.9)	
Marital status					0.189
Unmarried	819 (96.8)	501 (96.2)	184 (96.8)	134 (99.3)	
Married	27 (3.2)	20 (3.8)	6 (3.2)	1 (0.7)	
Unemployment					0.857
No	763 (90.2)	471 (90.4)	172 (90.5)	120 (88.9)	
Yes	83 (9.8)	50 (9.6)	18 (9.5)	15 (11.1)	
Income level, monthly					0.001
Low (< NT 40,000)	435 (51.4)	251 (48.2)	95 (50)	89 (65.9)	
High(≥NT 40,000)	411 (48.6)	270 (51.8)	95 (50)	46 (34.1)	
**BMI (kg/m^2^)**					0.274
Underweight (<18.5)	35 (4.1)	25 (4.8)	6 (3.2)	4 (3)	
Normal (18.5–24.9)	550 (65.0)	328 (63)	124 (65.3)	98 (72.6)	
Overweight (≥25)	261 (30.9)	168 (32.2)	60 (31.6)	33 (24.4)	
**Current smoker**					0.791
No	511 (60.4)	319 (61.2)	111 (58.4)	81 (60)	
Yes	335 (39.6)	202 (38.8)	79 (41.6)	54 (40)	
**Alcohol consumption**					0.191
No	468 (55.3)	301 (57.8)	98 (51.6)	69 (51.1)	
Yes	378 (44.7)	220 (42.2)	92 (48.4)	66 (48.9)	
**Recreational Drug Use in the Past 3 Months**					<0.001
No	724 (85.6)	469 (90)	154 (81.1)	101 (74.8)	
Yes	122 (14.4)	52 (10)	36 (18.9)	34 (25.2)	
**Living status**					0.063
Alone	383 (45.3)	241 (46.3)	89 (46.8)	53 (39.3)	
With others	463 (54.7)	280 (53.7)	101 (53.2)	82 (60.7)	
**Disclosure of disease to**					
Family members					0.721
No	463 (54.7)	290 (55.7)	103 (54.2)	70 (51.9)	
Yes	383 (45.3)	231 (44.3)	87 (45.8)	65 (48.1)	
Friends					0.11
No	785 (92.8)	491 (94.2)	171 (90)	123 (91.1)	
Yes	61 (7.2)	30 (5.8)	19 (10)	12 (8.9)	
Lover					0.035
No	292 (34.5)	193 (37)	65 (34.2)	34 (25.2)	
Yes	554 (65.5)	328 (63)	125 (65.8)	101 (74.8)	
**Having a Lover**					0.089
No	498 (58.9)	298 (57.2)	109 (57.4)	91 (67.4)	
Yes	348 (41.1)	223 (42.8)	81 (42.6)	44 (32.6)	
**CES-D**					0.082
≥16	335 (39.6)	196 (37.6)	74 (38.9)	65 (48.1)	
<16	511 (60.4)	325 (62.4)	116 (61.1)	70 (51.9)	
**HBV**					0.42
No	787 (93.0)	481 (92.3)	177 (93.2)	129 (95.6)	
Yes	59 (7.0)	40 (7.7)	13 (6.8)	6 (4.4)	
**HCV**					0.835
No	786 (92.9)	486 (93.3)	176 (92.6)	124 (91.9)	
Yes	60 (7.1)	35 (6.7)	14 (7.4)	11 (8.1)	
**History of sexual transmitted disease acquired**					0.383
No	297 (35.1)	192 (36.9)	60 (31.6)	45 (33.3)	
Yes	549 (64.9)	329 (63.1)	130 (68.4)	90 (66.7)	
**CD4 count, cells/mm^3^**					0.018
<200	14 (1.7)	5 (1)	3 (1.6)	6 (4.4)	
≥200	832 (98.3)	516 (99)	187 (98.4)	129 (95.6)	
**Viral load, copies/mL**					<0.001
Undetectable	767 (90.7)	495 (95)	172 (90.5)	100 (74.1)	
Detectable	79 (9.3)	26 (5)	18 (9.5)	35 (25.9)	
**STR**					0.088
No	154 (18.2)	85 (16.3)	36 (18.9)	33 (24.4)	
Yes	692 (81.8)	436 (83.7)	154 (81.1)	102 (75.6)	

SD, standard deviation; BMI, Body mass index; HBV, Hepatitis B virus; HCV, Hepatitis C virus; year, yr

**Table 2:** Univariate and multivariate analyses of factors associated with PWH.

	PWH with Unintentional Non-Adherence (Versus Full Adherence)				PWH with Intentional Non-Adherence (Versus Full Adherence)			
	Univariate		Multivariate (Backward)		Univariate		Multivariate (Backward)	
	Odds Ratio (95% CI)	*p* Value	Adjusted Odds Ratio (95% CI)	*p* Value	Odds Ratio (95% CI)	*p* Value	Adjusted Odds Ratio (95% CI)	*p* Value
**Socio-demographics**								
Age, yr	0.978 (0.960, 0.997)	0.024	0.981 (0.962, 0.999)	0.048	0.950 (0.928,0.973)	<0.001	0.969 (0.939, 1.000)	0.048
Diagnosed, yr	0.967 (0.938, 0.007)	0.031			0.943 (0.909, 0.978)	0.001		
Treatment duration, yr	0.977 (0.948, 1.007)	0.133			0.966 (0.933,1.001)	0.054		
Sex								
Female	1				1			
Male	0.911 (0.175, 4.735)	0.912			0.644 (0.124, 3.358)	0.602		
Sex orientation								
Non-Homosexual	1				1			
Homosexual	1.133 (0.535,2.400)	0.745			1.133 (0.535, 2.400)	0.745		
Education level completed								
≤High school	1				1			
University or above	0.702 (0.479, 1.029)	0.07			0.586 (0.384, 0.893)	0.013		
Marital status								
Unmarried	1				1			
Married	0.817 (0.323, 2.066)	0.669			0.187 (0.25. 1.406)	0.103		
Unemployment								
No	1				1			
Yes	0.986 (0.560, 1.737)	0.986			1.177 (0.639, 2.169)	0.6		
Income level, monthly								
Low (<NT 40,000)	1				1		1	
High(≥NT 40,000)	0.930 (0.667, 1.296)	0.667			0.480 (0.324, 0.713)	<0.001	0.583 (0.379, 0.896)	0.014
BMI (kg/m^2^)								
Underweight (<18.5)	1				1			
Normal (18.5–24.9)	1.575 (0.631, 3.932)	0.33			1.846 (0.635, 5.495)	0.257		
Overweight (≥25)	1.488 (0.582, 3.804)	0.406			1.228 (0.401, 3.761)	0.72		
**Current smoker**								
No	1				1			
Yes	1.124 (0.802, 1.576)	0.498			1.053 (0.715, 1.550)	0.794		
**Alcohol consumption**								
No	1				1			
Yes	1.284 (0.920, 1.793)	0.141			1.309 (0.895, 1.913)	0.165		
**Recreational Drug Use in the Past 3 Months**								
No	1		1		1		1	
Yes	2.108 (1.328, 3.348)	0.002	1.961 (1.228, 3.133)	0.005	3.036 (1.874, 4.920)	<0.001	2.920 (1.722, 4.951)	<0.001
**Living status**								
Alone	1				1			
with others	0.977 (0.700, 1.363)	0.89			1.332 (0.905, 1.959)	0.146		
**Disclosure of disease to**								
Family members								
No	1				1			
Yes	1.060 (0.760, 1.480)	0.73			1.166 (0.798, 1.703)	0.428		
Friends								
No	1				1			
Yes	1.819 (0.998, 3.315)	0.051			1.597 (0.794, 3.209)	0.189		
Lover								
No	1				1		1	
Yes	1.132 (0.799, 1.603)	0.487			1.748 (1.140, 2.680)	0.01	1.639 (1.036, 2.592)	0.035
**Having a Lover**								
No	1				1			
Yes	0.993 (0.710, 1.389)	0.968			0.646 (0.433, 0.963)	0.032		
**CES-D**								
≥16	1				1			
<16	0.945 (0.672, 1.330)	0.747			0.649 (0.444, 0.951)	0.027		
**HBV**								
No	1				1			
Yes	0.883 (0.462, 1.690)	0.708			0.559 (0.232, 1.348)	0.196		
**HCV**								
No	1				1			
Yes	1.105 (0.580, 2.102)	0.762			1.232 (0.608, 2.494)	0.563		
**History of sexual transmitted disease acquired**								
No	1				1			
Yes	1.264 (0.888, 1.801)	0.194			1.167 (0.783, 1.741)	0.448		
**CD4 count, cells/mm^3^**								
<200	1				1			
≥200	0.604 (0.143, 2.552)	0.493			0.208 (0.063, 0.693)	0.011		
**Viral load, copies/mL**								
Undetectable	1				1		1	
Detectable	1.992 (1.006, 3.724)	0.031			6.663 (3.840, 11.562)	<0.001	5.696 (3.155, 10.284)	<0.001
**STR**								
No	1				1		1	
Yes	0.834 (0.542, 1.283)	0.409			0.603 (0.382, 0.951)	0.03	0.452 (0.269, 0.760)	0.003

confidence interval, CI; year, yr

**Table 3:** Self-Reported Reasons for Unintentional and Intentional ART Non-Adherence Among PWH.

Adherence Category	Reason for Non-Adherence	Number of Respondents (*n*)	Percentage (%)
Unintentional non-adherence (*n* = 190)	Being busy with work/life	102	53.68%
	Forgot to bring medication	30	15.79%
	Missed dose due to sleep	27	14.21%
	Forgot whether medication was taken	14	7.37%
	Daily routine did not match medication schedule	9	4.74%
	Forgot to take medication due to alcohol use	9	4.74%
	Forgot to take medication due to recreational drug use	9	4.74%
	Fatigue from work/life	7	3.68%
	Forgot to refill prescription	2	1.05%
	Forgot after turning off alarm	2	1.05%
	Poor memory	2	1.05%
	Did not know how to take the medication	2	1.05%
	Too many medications to remember	1	0.53%
	Zoning out	1	0.53%
	Forgot due to appendicitis	1	0.53%
	total	218	
Intentional non-adherence (*n* = 135)	Ran out of medication	25	18.52%
	Experienced side effects	20	14.81%
	In poor emotional state	20	14.81%
	Did not want to take medication	17	12.59%
	Felt healthy and chose not to take medication	7	5.19%
	Intentionally skipped due to recreational drug use	6	4.44%
	Afraid taking medication would reveal HIV status	4	2.96%
	Too many medications	2	1.48%
	skipped medication due to fasting or not eating	2	1.48%
	Intentionally skipped due to alcohol use	2	1.48%
	Medication was switched	1	0.74%
	Medication had an unpleasant taste	1	0.74%
	Taking medication reminded them of having HIV	1	0.74%
	Do not answer or not having specific reasons	43	31.85%
	total	151	

Note: Participants were allowed to report multiple reasons for non-adherence. Percentages are based on total number of reasons provided within each group. ART = antiretroviral therapy; PWH = people with HIV.

## Data Availability

The data presented in this study are not publicly available due to privacy and ethical restrictions.

## Abbreviations

The following abbreviations are used in this manuscript:

ART	Antiretroviral therapy
ADIs	AIDS-defining illnesses
CES-D	Center for Epidemiologic Studies Depression Scale
MARS-5	The Medication Adherence Report Scale
PWH	People with HIV
